# Reviewed article: Damasceno LLV, Sousa GJB, Garces TS, Cestari VRF, Araujo JCM, Moreira TMM, Pereira MLD. Spatial and temporal patterns and factors associated with mortality from Chagas’ disease: an ecological study, Ceará, 2002-2022. Epidemiol Serv Saude. 2025:34;e20240852

**DOI:** 10.1590/S2237-96222025v34e20240852.a

**Published:** 2025-08-08

**Authors:** Fabio Zicker

**Affiliations:** FIOCRUZ, CDTS

**Table:** 

Rating	Excellent	Good	Average	Below average	Poor
Interest		✓			
Quality	✓				
Originality		✓			
Overall		✓			

**Table:** 

Questionnaire	Yes	No	Not applicable
Does the manuscript contain new and significant information to justify publication?	✓		
Does the Abstract (Summary) clearly and accurately describe the content of the article?	✓		
Is the problem significant and concisely stated?	✓		
Are the methods described comprehensively?	✓		
Are the interpretations and conclusions justified by the results?	✓		
Is adequate reference made to other work in the field?	✓		

## Manuscript Structure

Length of article is: Adequate

Number of tables is: Adequate

Number of figures is: Adequate

Please state any conflict(s) of interest that you have in relation to the review of this paper (state “none” if this is not applicable): None

## Comments

Congratulations on a well-written interdisciplinary article using cutting-edge spatial and statistical modeling addressing inequalities in Chagas disease mortality in Ceará. The study is methodologically sound and presents a valuable contribution to public health policy. 

